# Frequency, risk factors, and complications of induced abortion in ten districts of Madagascar: results from a cross-sectional household survey

**DOI:** 10.1186/s12905-020-00962-2

**Published:** 2020-05-06

**Authors:** Rila Ratovoson, Amber Kunkel, Jean Pierre Rakotovao, Dolores Pourette, Chiarella Mattern, Jocelyne Andriamiadana, Aina Harimanana, Patrice Piola

**Affiliations:** 1 Epidemiology and Clinical Research Unit, Institut Pasteur of Madagascar, BP 1274 Ambatofotsikely Avaradoha, 101, Antananarivo, Madagascar; 2 Emerging Diseases Epidemiology Unit, Institut Pasteur, Paris, France; 3 Maternal and Child Survival Program (MCSP), Antananarivo, Madagascar; 4 Centre Population et Développement (UMR 196, IRD, Université Paris Descartes), Paris, France; 5 SageSud ERL INSERM 1244, Paris, France; 6 United States Agency for International Development Madagascar, Antananarivo, Madagascar; 7 Epidemiology and Public Health Unit, Institut Pasteur of Cambodia, Phnom Penh, Cambodia

**Keywords:** Unsafe abortion, Induced abortion, Survey, Incidence, Family planning, Contraception, Madagascar

## Abstract

**Background:**

Madagascar has restrictive abortion laws with no explicit exception to preserve the woman’s life. This study aimed to estimate the incidence of abortion in the country and examine the methods, consequences, and risk factors of these abortions.

**Methods:**

We interviewed 3179 women between September 2015 and April 2016. Women were selected from rural and urban areas of ten districts via a multistage, stratified cluster sampling survey and asked about any induced abortions within the previous 10 years. Analyses used survey weighted estimation procedures. Quasi-Poisson regression was used to estimate the incidence rate of abortions. Logistic regression models with random effects to account for the clustered sampling design were used to estimate the risk of abortion complications by abortion method, provider, and month of pregnancy, and to describe risk factors of induced abortion.

**Results:**

For 2005–2016, we estimated an incidence rate of 18.2 abortions (95% CI 14.4–23.0) per 1000 person-years among sexually active women (aged 18–49 at the time of interview). Applying a multiplier of two as used by the World Health Organization for abortion surveys suggests a true rate of 36.4 per 1000 person-year of exposure. The majority of abortions involved invasive methods such as manual or sharp curettage or insertion of objects into the genital tract. Signs of potential infection followed 29.1% (21.8–37.7%) of abortions. However, the odds of potential infection and of seeking care after abortion did not differ significantly between women who used misoprostol alone and those who used other methods. The odds of experiencing abortion were significantly higher among women who had ever used contraceptive methods compared to those who had not. However, the proportion of women with a history of abortion was significantly lower in rural districts where contraception was available from community health workers than where it was not.

**Conclusions:**

Incidence estimates from Madagascar are lower than those from other African settings, but similar to continent-wide estimates when accounting for underreporting. The finding that the majority of abortions involved invasive procedures suggests a need for strengthening information, education and communications programs on preventing or managing unintended pregnancies.

## Background

The World Health Organization (WHO) defines unsafe abortion as “a procedure for terminating an unwanted pregnancy either by persons lacking the necessary skills or in an environment lacking the minimal medical standards (less safe), or both (least safe)” [1]. Each year from 2010 to 2014, around 25 million unsafe abortions occurred worldwide, most of which (97%) occurred in developing countries. The proportion of unsafe abortions was highest in countries with highly restrictive abortion laws: 13% of all abortions in countries in which abortion was legal were unsafe, compared with 75% in countries where abortion was completely banned or allowed only to save the woman’s life or physical health [2–4].

Women in Africa are particularly at risk of dying from unsafe abortions, suggesting that use of dangerous invasive methods by untrained individuals is common [2, 4]. Most studies on abortions in African settings rely on hospital data, whether directly from women with complications or using health records. Incidence varies between countries, as do the laws governing abortion. For example, in 2012–2013, studies based at healthcare facilities estimated the abortion rate at 17 per 1000 women aged 15–44 in Senegal; 33 per 1000 women aged 15–49 in Nigeria; 36 per 1000 women aged 15–49 in Tanzania; and 48 per 1000 women aged 15–49 in Kenya [5–8]. All four countries have restrictive abortion laws, most frequently limiting abortions to cases in which the woman’s life is at risk [5–8].

The most serious complication of unsafe abortion remains death. In addition, serious hemorrhages, pelvic inflammatory disease (which may be caused by uterine perforation), and infection are also encountered as complication of unsafe abortion in health facilities in countries including Madagascar [9–11].

The law in Madagascar is especially restrictive and targets all women who have abortions for any reason, as well as any individuals who assist women in obtaining them [12, 13]. The law was reiterated in 2017 through one that established general rules on reproductive health and family planning in Madagascar (article 27 of the law n°2017–043) [14]. Despite this, the Ministry of Public Health estimated that 11.8% of maternal deaths in 2012 were attributed to complications of unsafe abortion [15]. There are, however, no estimates of the population-level incidence of induced abortions in the country [16]. Furthermore, published studies on risk factors, providers, and methods of abortion in Madagascar are available only from surveys in healthcare facilities among women seeking abortions or post-abortion care [9, 17]. The risk factors for induced abortion found in previous studies were the state of women’s health, socio-economic and cultural factors [9]. Other factors such as gaps in sex education, forced sex, the social stigma of pregnancy outside marriage, inappropriate use of contraceptives, and irresponsibility of the father of the child can also lead to induced abortion [17]. However, facility-based surveys may be insufficiently representative of abortions in the population, as not all women with abortion complications seek medical care, either for fear of being reported to the judicial authorities or for financial reasons [10, 17, 18]. In particular, women in rural areas who have limited access to healthcare may turn to traditional birth attendants or self-treat both for abortions and post-abortion care.

The primary aim of this study was to estimate the incidence of abortion in Madagascar and examine variations in women’s experiences of abortion and related complications by various socio-demographic characteristics. Because of the increased availability of family planning services in Madagascar since the 2000s, we also examined variations in the likelihood of having an abortion by contraceptive use history (non-use and use of less or highly effective methods). Understanding the incidence and nature of abortions in a highly restrictive environment such as that of Madagascar is important for informing policies and programs to improve women’s health outcomes when they experience unintended pregnancies in such settings.

## Methods

A cross-sectional, population-based survey of women aged 18–49 was conducted to estimate the frequency, risk factors, and complications of induced abortion in 10 selected districts of Madagascar. Women < 18 years were not included due to concerns about asking for consent from the parents; however, information was sought about all abortions occurring in the last 10 years and thus captured abortions occurring during adolescence among women aged 29 years and below. A multistage cluster sampling scheme was used to select women for inclusion in the study.

### Sampling procedure

Ten districts were chosen to be included in this study (Table S1). These districts were purposively selected to have varying geographical distribution (representing northern, southern, eastern and western areas of Madagascar) and presence (or not) of community health workers (CHWs) trained to provide family planning services.

For nine of these districts, the district capital was selected as the urban area to be included in the study, as well as a rural area defined as all rural communes with at least 80% of their surface area falling inside a 50 km radius of the selected urban area. For the district of Antananarivo, only an urban area was selected.

**Fig. 1:**
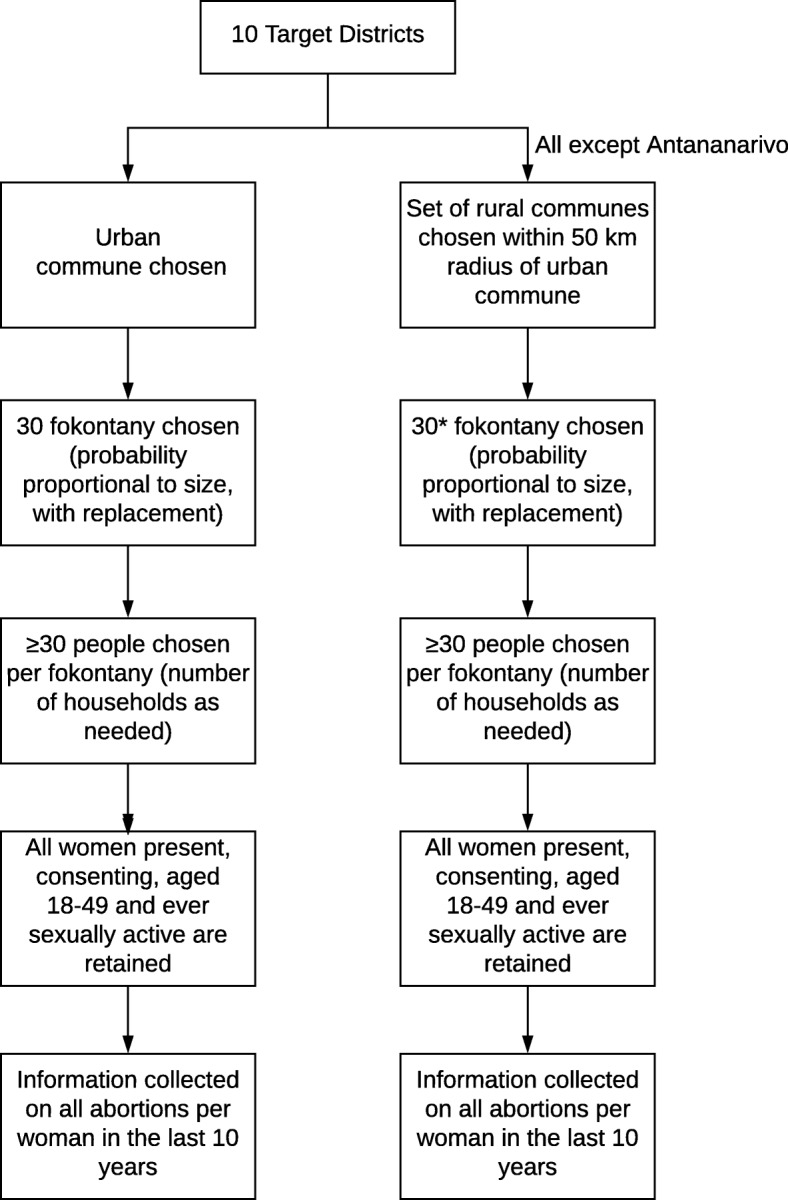
Sampling Scheme of participants in abortion in women aged between 18 and 49 years in 10 districts in Madagascar (2015–2016). Thirty fokontany were chosen per district, with the exception of the rural areas of Sambava [19] and Vohemar [20]. The number of people selected per fokontany sample ranged from 28 to 56 (target: at least 30)

A multi-stage cluster sampling approach was used to select women for this study (Fig. 1). First, strata (referred to here as “regions”) were defined by district and urban/rural areas, and were purposively selected as described above (19 strata in total). Within each region, approximately 30 fokontany (villages) were selected with replacement with probability proportional to population size. Extenuating circumstances such as insecurity or inaccessibility led to some originally selected fokontany being replaced with backup options, which may limit the applicability of the results to the most remote or insecure areas of the included districts. This affected 35 of the originally chosen fokontany, ranging from 0 in Antananarivo and Moramanga to 12 in rural Mitsinjo. Following the reassignments, 33 fokontany were ultimately chosen in the final sample for Vohemar and 27 for Sambava, two neighboring districts in the north of the country. All data collection occurred between September 1st 2015 and April 8th 2016; because we asked about all abortions in the past 10 years, the reported abortions could have occurred between 2005 and 2016.

A map of each selected fokontany was drawn to guide the random selection of homes. On the map, the center of the fokontany was located and a line drawn straight east from the center to the fokontany border. Points were then added dividing the line into 9 equal distances and a number between 1 and 9 was drawn at random. The home located at or closest to the east of the selected point was chosen as the first home to be interviewed. Subsequent homes were chosen by successively choosing the next closest home on the interviewer’s right upon exiting the home.

Within each home, information was requested on the number, age, and sex of all inhabitants, and all women aged 18–49 were invited to participate in the study. When women were absent, interviewers returned to the home at a different day or time to repeat the attempted contact; women who were repeatedly absent were not included in the study.

### Number of subjects

The target number of individuals to be enumerated was set to at least 17,100 people (19 regions with 30 fokontany each with at least 30 individuals each). Assuming that 17% of these individuals were women of the target age [21], this would result in 2907 women aged 18–49 being assessed for inclusion. If 20% of these women either were absent, did not provide consent, or were not sexually active, a total of 2325 women would be included. Assuming an unsafe abortion rate estimates at 0.03/year [22], and 10 years of follow-up per women, a total of 602 abortions would be expected. With a design effect of 2.0 and a risk of abortion complications of 25%, the precision around this estimate would be approximately 5%.

Because fokontany were selected with replacement, some fokontany were selected to be included more than once. Dividing the number of people enumerated per fokontany by the number of times that fokontany was selected, the number of people sampled in each fokontany sample varied from 28 to 56 depending on how many individuals the interviewers were able to enumerate in the available time.

### Interviews

Women in the target age range who were present during at least one interviewer visit and who provided informed consent were interviewed by trained female social workers. Interviews captured demographic information, reproductive history, and knowledge and use of contraception. Information on household assets and amenities was also collected and used to create an index for socioeconomic status (SES) using uncentered principal components analysis [23, 24]. To minimize the risk of psychosocial distress to the participants, interviews began with information about the composition of household and the socioeconomic status before moving on to more sensitive questions about past pregnancies, abortions, and use of family planning. Women who reported having ever been sexually active were asked to recall all abortions, either spontaneous or induced, in the last 10 years.

This paper reports the results for induced abortions only. Further information and sensitivity analyses on the definition of induced abortion are included in the Supplement.

### Statistical analysis

Population estimates of the frequency of abortion, methods and providers, and complications were weighted based on the sampling and response probabilities of each included woman and calculated using the R package “survey” for complex survey analyses. A survey weighted quasi-Poisson regression was used to estimate the incidence rate of induced abortions. For this calculation, the dependent variable was the number of abortions in the last 10 years per woman and we specified an offset term as the natural log of the minimum of either 10 years or the number of years since initiation of sexual activity. The incidence rate was taken to be the exponent of the intercept term.

Logistic mixed effects models using the R function glmer from package lme4 were used to explore associations between individual-level factors (independent variables) and occurrence of an abortion within the last 10 years (binary dependent variable). The models were also used to explore variation in potential infections and seeking care for symptoms following an abortion by gestational age of pregnancy at the time of abortion, abortion provider, and abortion method. Additional details about the statistical methods and the variables included in the regression analysis are provided as supplementary material.

### Ethical approval and consent to participate

This study was approved by the Ethics Committee of the Ministry of Public Health of Madagascar (N°051-MSANP/CE - 05/05/2015). At the household level, the fieldworkers explained in the local language that they were conducting a survey on maternal health that would contain sensitive questions about past pregnancies, abortions, and use of family planning. Verbal consent was obtained from the head of the household (or his wife / her husband). At this point, the fieldworkers asked about whether any women in the target age range were present in the home. Any eligible women were given a more detailed explanation of the study as well as information about confidentiality, privacy and the right to refuse to participate or withdraw before conducting any interview. Interviews only occurred if the woman agreed to participate and signed an informed consent form. In rare cases, if the woman requested it, other household members were allowed to remain for the portion of the interview related to household composition and socioeconomic status, but questions about pregnancy history and use of family planning began only when the interviewer was alone with the woman.

## Results

### Frequency of induced abortions

**Fig. 2:**
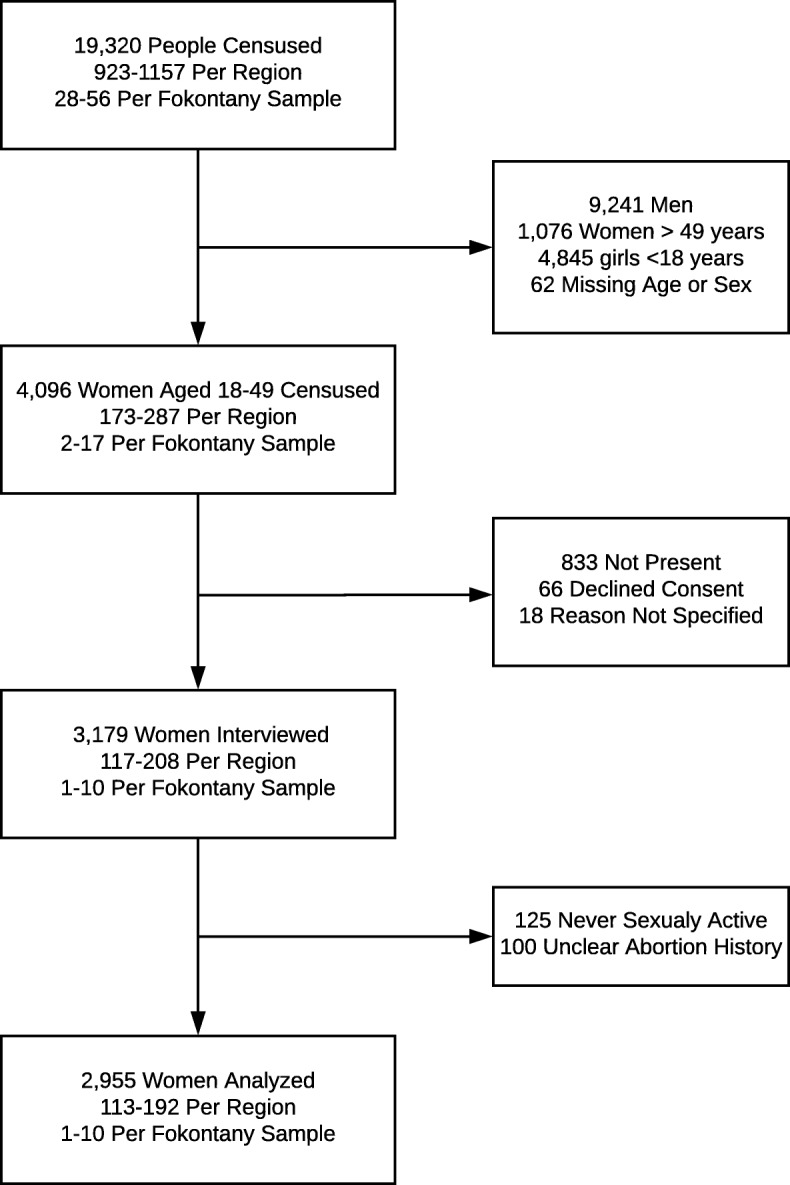
Flowchart of included participants in abortion in women aged between 18 and 49 years in 10 districts in Madagascar (2015–2016)

In total, 19,320 people were enumerated, of whom 4096 were women aged 18–49, 3179 were interviewed, and 2955 women were retained in the analysis (Fig. 2). A total of 459 induced abortions in the last 10 years were reported by 352 women.

Applying survey weights to account for unequal population sizes of the different regions, we estimated that 11.0% (95% CI 8.5–14.2%) of sexually active women aged 18–49 in the study area had at least one induced abortion in the last 10 years. This proportion varied considerably based on location, from 2.0% (0.5–8.4%) in rural areas of Toliara to 25.2% (16.9–35.9%) in urban areas of Sambava (Table 1).

**Table 1:** Proportion with at least one induced abortion in the last 10 years among women aged between 18 and 49 years in 10 districts in Madagascar (2015–2016)

District	Area	Estimated percentage of women with abortions in last 10 years
Ambovombe	Urban	4.8% (1.7, 12.9)
	Rural (Without FP^a^)	5.1% (1.8, 13.6)
Antananarivo	Urban	9.7% (4.9, 18.4)
Mahajanga	Urban	25.1% (18.5, 33.0)
	Rural (Without FP)	16.6% (11.3, 23.9)
Maroantsetra	Urban	18.1% (13.2, 24.3)
	Rural (Without FP)	8.7% (5.5, 13.4)
Mitsinjo	Urban	4.4% (1.8, 10.4)
	Rural (With FP)	9.6% (5.3, 16.6)
Moramanga	Urban	7.3% (3.7, 14.0)
	Rural (With FP)	2.5% (0.8, 8.1)
Sambava	Urban	25.2% (16.9, 35.9)
	Rural (Without FP)	9.7% (5.3, 17.1)
Toamasina	Urban	17.4% (12.5, 23.7)
	Rural (With FP)	4.2% (2.0, 8.5)
Toliara	Urban	18.8% (13.8, 25.1)
	Rural (With FP)	2.0% (0.5, 8.4)
Vohemar	Urban	21.7% (14.8, 30.6)
	Rural (With FP)	6.3% (3.4, 11.5)

^a^“Without FP” means family planning is (not) available from community health workers in the district. “With FP” means family planning is available from community health workers in the district

Of those women who reported at least one abortion in the last 10 years, 79.2% (69.0–86.7%) reported only one, 16.7% (10.1,26.4%) reported two, and 4.1% (2.3, 7.4%) reported three or more. We estimated an incidence rate of 18.2 (14.4–23.0) abortions per 1000 person-years at risk. Most of these abortions occurred early in pregnancy (Fig. S1). To adjust for underreporting due to self-report, WHO applies an augmenting factor of 2 to estimates obtained from abortion surveys [25], which would suggest a true rate of 36.4 per 1000 person-years at risk.

### Abortion methods and providers

Table 2 presents the methods and providers that women described for each induced abortion reported in the study. Many women reported multiple methods and providers for the same abortion. We estimated the proportion of women who used misoprostol alone (orally, vaginally, or both) at 16.0% (10.7–23.3%). We estimated that 63.0% (52.8–72.1%) of women saw a qualified medical provider (doctor, nurse or midwife) to perform their abortion. Of those women who saw only a qualified provider, 61.0% (48.1–72.5%) received curettage, 39.1% (29.8–49.3%) had insertion of a catheter or stem into the genital tract, and only 10.0% (4.5–20.7%) received misoprostol alone.

**Table 2:** Abortion methods and providers reported by women aged between 18 and 49 years in 10 districts in Madagascar (2015–2016)

Method	Percentage of Abortions
Oral misoprostol	25.2% (18.0–34.0)
Vaginal misoprostol	6.9% (3.3–14.1)
Contraceptive pills	9.2% (4.3–18.4)
Curettage^a^	42.2% (33.1–51.9)
Insertion of a tube or plant stem into the genital tract	29.3% (20.6–39.9)
Ingestion of a herbal decoction (tambavy)	12.8% (8.3–19.3)
Other^b^	22.5% (16.5–29.8)
**Provider**	
Doctor	43.7% (32.0–56.1)
Nurse or midwife	23.9% (16.8–32.7)
Traditional healer or birth attendant (“matrone”)	4.9% (1.7–13.6)
Self-administered	19.4% (12.8–28.4)
Other^c^	13.0% (7.2–22.3)

^a^Curettage also includes manual cleaning of the uterus (“curage”)^. b^Other methods included injections, antimalarial pills, alcohol or vinegar, massage, and “unknown”. Only one woman reported receiving vacuum aspiration. ^c^Other providers, when specified, included family members, friends, and pharmacists; it also includes those who listed none of the above possibilities

### Symptoms of induced abortions

We estimated that 60.6% (52.1–68.4%) of abortions resulted in at least one symptom or complication (Table 3). The most frequent symptom reported was hemorrhage or blood clots. Nearly one third of abortions led to signs of potential infection (which we defined as fever, chills, or foul smelling vaginal discharge).

**Table 3:** Abortion symptoms and complications among women aged between 18 and 49 years in 10 districts in Madagascar (2015–2016)

Symptoms	Percentage of abortions
Hemorrhage or blood clots	46.3% (37.8–55.0)
Dizziness or confusion	31.9% (23.9–41.0)
Abdominal pain	27.7% (17.9–40.1)
Foul smelling vaginal discharge	19.7% (12.6–29.4)
Fever or chills	18.1% (11.0–28.2)
Possible infection (fever, chills, or foul smelling vaginal discharge)	29.1% (21.8–37.7)

Results from logistic regression analysis examining variations in abortion-related infections showed that the likelihood of experiencing such infections was significantly greater among women at late than those at early gestational age of pregnancy (OR: 1.37, 95% CI 1.06–1.76, *p* = 0.01). The results further showed that the likelihood of experiencing infection was not significantly associated with abortion method (misoprostol alone vs. other; OR = 0.92, 95% CI: 0.44–1.95, *p* = 0.84) or provider (qualified medical provider alone vs. other; OR = 0.70, 95% CI: 0.39–1.25, *p* = 0.22).

### Care seeking after abortion

Table S3 shows women’s care seeking behavior for complications after an abortion. All women with abortions were asked whether they sought care for complications resulting from the abortion. We estimated that 27.7% (21.8–34.6%) of all abortions result in women seeking care for complications, though only 2.4% (1.1–5.4%) result in hospitalization. Women who sought care for complications more often consulted private hospitals and clinics than public hospitals and health centers (mean difference in probability of consulting public versus private provider = − 0.29, 95% CI (− 0.52, − 0.05), *p* = 0.02 by survey weighted paired t-test).

Results from logistic regression analysis examining variations in care-seeking after an abortion showed that the likelihood of seeking care was significantly greater among women at late than those at early gestational age of pregnancy (OR: 1.27, 95% CI 1.05–1.54, *p* = 0.01). The results further showed that the risk of infection was not significantly associated with abortion method (misoprostol alone vs. other; OR = 1.50, 95% CI: 0.81–2.77, *p* = 0.20) or provider (qualified medical provider alone vs. other; OR = 1.34, 95% CI: 0.82–2.20, *p* = 0.24).

### Factors associated with history of abortion

Table 4 shows the results of logistic regression analyses examining variations in the likelihood of experiencing an induced abortion in the last 10 years by women’s background characteristics. We report both unadjusted estimates (controlling for setting, i.e. district, fokontany, and urban/rural and years at risk only to account for the study design) and adjusted estimates (controlling for setting, years at risk, and all other variables in the table).

**Table 4:** Odds ratios from logistic regression analysis examining variations in the likelihood of experiencing an abortion in the last 10 years among women 18–49 years in 10 districts in Madagascar, 2015–2016

		Unadjusted estimates^b^		Adjusted estimates^c^	
Variable	Values	Odds ratio (95% CI)	*p*-value	Odds ratio (95% CI)	p-value
Age	≥35	Ref	Ref	Ref	Ref
	25 to < 35	2.87 (2.11, 3.92)	< 0.0001*	2.52 (1.83, 3.48)	< 0.0001*
	20 to < 25	4.16 (2.53, 6.85)	< 0.0001*	3.95 (2.33, 6.66)	< 0.0001*
	< 20	5.50 (2.63, 11.49)	< 0.0001*	5.52 (2.53, 12.02)	< 0.0001*
Maximum Education	Primary school or less	Ref	Ref	Ref	Ref
	Middle school	1.87 (1.41, 2.47)	< 0.0001*	1.62 (1.18, 2.13)	0.002*
	High school or more	2.61 (1.87, 3.64)	< 0.0001*	2.59 (1.71, 3.72)	< 0.0001*
Religion	Catholic	Ref	Ref	Ref	Ref
	Church of Jesus Christ in Madagascar (FJKM)	0.92 (0.69, 1.23)	0.59	0.91 (0.68, 1.23)	0.56
	Other Christian	1.29 (0.95, 1.75)	0.11	1.35 (0.98, 1.86)	0.07
	Other (including Muslim, traditional religions)	0.57 (0.38, 0.86)	0.007*	0.74 (0.49, 1.13)	0.17
Civil Status	Single	Ref	Ref	Ref	Ref
	Married or living with a partner	0.67 (0.51, 0.89)	0.006*	0.77 (0.56, 1.05)	0.10
	Other (Widowed, Divorced)	0.60 (0.38, 0.93)	0.02*	0.74 (0.46, 1.20)	0.22
SES^a^	Quintile 1	Ref	Ref	Ref	Ref
	Quintile 2	0.76 (0.52, 1.11)	0.15	0.71 (0.48, 1.06)	0.09
	Quintile 3	0.80 (0.55, 1.17)	0.25	0.71 (0.48, 1.06)	0.10
	Quintile 4	1.07 (0.74, 1.54)	0.72	0.99 (0.68, 1.46)	0.98
	Quintile 5	1.21 (0.85, 1.73)	0.28	0.97 (0.66, 1.43)	0.88
Transactional Sex	Never	Ref	Ref	Ref	Ref
	Ever	1.53 (1.19, 1.96)	0.0008*	1.58 (1.21, 2.06)	0.0007*
Number of Live Births	0 live births	Ref	Ref	Ref	Ref
	≥1live birth	0.86 (0.61, 1.21)	0.39	1.07 (0.72, 1.60)	0.73
Ideal Number of Children	< 4	Ref	Ref	Ref	Ref
	≥4	0.67 (0.52, 0.86)	0.002*	0.84 (0.63, 1.10)	0.20
Contraceptive Use	No history of contraceptive use	Ref	Ref	Ref	Ref
	History of using less effective methods only	2.20 (1.41, 3.44)	0.0005*	1.75 (1.10, 2.79)	0.02*
	History of using more effective methods only	2.17 (1.55, 3.04)	< 0.0001*	1.91 (1.35, 2.71)	0.0003*
	History of using both more and less effective methods	4.48 (2.92, 6.87)	< 0.0001*	3.68 (2.35, 5.77)	< 0.0001*

^a^Quintiles were defined based on all women interviewed, including those excluded from the main analyses for unclear abortion history or never being sexually active. ^b^ Controlling for time at risk and location only. ^c^Controlling for all other variables in the table *p < 0.05

Individual-level variables analyzed for any possible association with history of abortion is provided in the supplementary files (Table S2).

In both unadjusted and multivariate analyses, a history of abortion was significantly more common among younger women (25- < 35 years, 20- < 25 years, and < 20 years compared to 35+ years, with higher odds ratios for lower age groups), women with higher levels of schooling (those with at least middle-level compared to those with primary-level education), and women who reported ever having sexual relations in exchange for money or gifts (“transactional sex”) compared to those who did not. In the unadjusted model, the likelihood of ever having an abortion was significantly lower among non-Christian (including those belonging to Muslim and traditional religions) than among Catholic women. It was also significantly lower among women who were (at the time of the interview) partnered, widowed, or divorced than among those who were single (never married), and among those who wanted four or more children than among those who wanted fewer than four children. These associations were, however, not statistically significant in the multivariate model. Abortion history was not significantly associated with SES or number of live births.

In both unadjusted and multivariate analyses, women who reported ever using contraceptive methods were also more likely to report a history of abortion compared to those who did not (Table 4). The association was similar for more effective and less effective methods, and strongest for women who reported a history of both. At the community level, however, women from rural areas of districts in which family planning was available from community health workers were more likely to report ever using more effective contraceptive methods, less likely to report ever using less effective methods, and less likely to report a history of abortion (Table S4).

## Discussion

We report the results from one of the largest community-based surveys of unsafe abortion in a country in which abortion is always illegal. Our results show that unsafe abortion is a public health problem in Madagascar, where abortions are frequently performed by invasive methods (manual or sharp curettage or insertion of stems or catheters into the genital tract) and can lead to serious health consequences including infections.

The incidence rate of abortions recorded for Madagascar (18.2 per 1000 person-years at risk) is lower than the average 34/1000 women per year which has been estimated for Africa overall [22], but similar to that estimated in Senegal, where abortion is also prohibited (17/1000 per year) [5]. It is worth noting that the results from Madagascar may not be directly comparable with other estimates when different methods have been used to derive them. Indirect methods of estimating abortion incidence are common; for example, the estimated value for Senegal was based on a survey of 168 health facilities and used an indirect method to estimate abortion incidence by multiplying the number of women treated in facilities for complications of induced abortion by the inverse of the probability that women who had had an abortion sought treatment for a complication. Applying the inflation factor of 2 used by WHO for estimates obtained from abortion surveys [25] to account for such underreporting would suggest a true rate of 36.4 per 1000 person-years at risk. Although the inflated rate is not directly observed in the study, it could be considered when comparing the results to those obtained from studies using different methodologies.

This study shows that abortion methods in Madagascar remain mostly invasive, with usage of misoprostol being uncommon relative to other settings like Latin America [26, 27]. In Africa, curettage was similarly preferred to Misoprostol in four Botswana hospitals [19]. The high use of invasive methods rather than misoprostol can be explained by the lack of knowledge of misoprostol in some African countries [28, 29]. Furthermore, based on our study findings it is not clear that misoprostol as currently used in Madagascar decreases complications, as use of misoprostol alone was not significantly associated with lower risk of potential infection or seeking care after abortion compared with other methods. A qualitative study that was carried out simultaneously revealed that the use of misoprostol is not controlled in Madagascar, with the interviewed women all reporting different dosages, none of which matched WHO guidelines [10]. Abortions performed by qualified medical personnel often involved curettage or insertion of objects into the genital tracts, and did not appear to result in fewer infections, suggesting that such personnel lacked the training or equipment necessary to ensure patient safety. Considering the frequency of abortions seen in this study despite the restrictive law, there is a need for formulating policies and guidelines for training Malagasy healthcare providers in post-abortion care, including provision of family planning services.

Although complications were reported by a majority of women with a history of abortion in this study, severe complications leading to hospitalization were rare. Such minor complications are not observed in hospital-based studies in which the vast majority of induced abortions are undocumented [30, 31]. The likelihood of experiencing complications serious enough to warrant seeking care increased with the gestational age at abortion. The proportion of women reporting an abortion before 12 weeks was 94% in our study, which is high compared to other African countries, for example 60% in Kenya and 80% in Nigeria [20, 32].

At the individual level, women who had ever used contraceptive methods were more likely to report a history of abortion compared to those who had never used a method. Another study conducted in Ivory Coast also found that women who reported using contraceptive methods were are more at risk of having an abortion [33]. As contraceptive use reflects women’s desire not to become pregnant, women who use contraceptives may be more likely to seek abortion in case of unwanted pregnancies or to use contraception following an abortion. Difficulties associated with contraceptive use, including poor compliance or discontinuation, could lead to unwanted pregnancies [34]. At the district level, districts in which contraception was available through CHWs had higher proportions of women who reported using more effective contraceptive methods, lower proportions of women who reported use of less effective methods, and lower proportions of women who reported history of abortion compared to those districts in which contraception was not available through CHWs. A study conducted in the 1990s attributed the rate of abortions in public hospitals in Madagascar (58.3/1000 live births) to inaccessibility of family planning services and low level of knowledge about modern contraceptive methods [35]. Since that time, use of modern contraceptive methods in Madagascar has increased (10% in 1997 versus 33% in 2013) [36]. The finding of high likelihood of using effective methods and low likelihood of experiencing an induced abortion in districts where contraception was available through CHWs suggests that providing contraception through this cadre may be an effective way of increasing knowledge and use of effective contraceptive methods.

Besides contraceptive use, other factors associated with increased risk of abortion included younger age, higher levels of education, and transactional sex (ever having sex in exchange for gifts or money). Non-Christian women were significantly less likely to have abortions compared to Catholic women in unadjusted but not multivariate analysis. The differing significance of these results in the different models could be due to confounding; however, the timing of the variables is unknown, the true causal structure is not possible to discern. Studies in other African settings found high likelihood of experiencing an abortion among Catholic women, those with higher levels of education, young women, and those who are single [20, 37]. The studies further show significant variations in the likelihood of having an abortion by wealth status [20, 37] and parity [20]. For instance, the prevalence of abortion was relatively high among nulliparous and non-poor women in Nigeria [20] as well as among wealthiest women in Ghana [37]. Urban areas had particularly high levels of abortion and unmet need for effective contraceptives. Variations by parity are difficult to determine in our dataset given that we did not ask about the timing at which abortions occurred. One interesting finding in our study was the significant association between transactional sex and abortion. The proportion of women reporting they had ever had transactional sex varied between regions and reached nearly 50% overall. The high proportions of women in the tourist regions of Sambava and Mahajanga who reported abortions suggests sex tourism may be a contributing factor.

There are some potential limitations in our analyses. The estimate for incidence of induced abortion that we obtained could be artificially low due to recall bias, as we asked women to describe abortions that occurred up to 10 years earlier, although induced abortions are not commonly forgotten [38]. In addition, women may have been reluctant to report abortions because of the sensitive nature of this topic. We sought to limit this bias by having the interviews conducted by trained social workers with experience asking about sensitive topics. Our inability to contact some women could also have led to bias. On the other hand, the health facility based methods used in most other African studies rely on strong assumptions about complications and care-seeking behavior. Fatal complications could not be captured in our study due its cross-sectional design. Women may also have been unable to remember minor abortion complications and to discern which outcomes, such as hemorrhage, were a feature of the abortion itself rather than a complication. Findings on variations in the history of abortion by contraceptive use could be affected by the time order of occurrence of the two events. However, it was not possible to determine which came first as we did not ask about the timing of occurrence of the two events. We also did not collect data on variables such as number of sexual partners that may increase use of both contraception and abortion.

## Conclusions

Despite the illegal nature of induced abortion, it continues to occur across Madagascar. Efforts have been made to facilitate access to contraceptive methods, for instance, through the passage of the Family Planning and Reproductive Health Act of 2017 [14, 15]. Our results showing low rates of abortion in districts where family planning services are available from CHWs suggest a need for further strengthening access to family planning in the country, especially among vulnerable subgroups. Invasive methods, including insertion of objects into the genital tract, were frequently used by women who reported induced abortions in this study. Further programs focused on information, education and communication should be strengthened on ways of avoiding or managing unintended pregnancies in Madagascar.

## Supplementary information

**Additional file 1.**

## Data Availability

The datasets used and/or analyzed during the current study are available from the corresponding author on reasonable request.

## Abbreviations

CHW: Community Health Worker
FP: Family Planning
SES: Socioeconomic status
WHO: World Health Organization
