# Supplementary material for: Frequency, risk factors, and complications of induced abortion in ten districts of Madagascar: results from a cross-sectional household survey
Source: BMC Womens Health. 2020 May 6;20:96. doi: 10.1186/s12905-020-00962-2 (PMC7203894; doi:10.1186/s12905-020-00962-2)
Supplement: Supplementary file 1 — Additional file 1. [file 12905_2020_962_MOESM1_ESM.docx]

Supplementary Material

**Frequency, risk factors, and complications of induced abortion in ten districts of Madagascar: a multistage, stratified cluster sampling survey**

**Sampling Procedures**

| District | Urban Population | Rural Area Included? | Rural Population |
| --- | --- | --- | --- |
| Ambovombe | 61,737 | Yes, without FP | 224,336 |
| Antananarivo | 1,247,025 | No | N/A |
| Mahajanga | 99,958 | Yes, without FP | 156,738 |
| Maroantsetra | 27,979 | Yes, without FP | 122,850 |
| Mitsinjo | 8,933 | Yes, with FP | 8,172 |
| Moramanga | 36,641 | Yes, with FP | 24,318 |
| Sambava | 41,672 | Yes, without FP | 254,447 |
| Toamasina | 263,661 | Yes, with FP | 175,561 |
| Toliara | 150,430 | Yes, with FP | 151,449 |
| Vohemar | 18,352 | Yes, with FP | 76,785 |

**Table S1: Districts included in the study**. The urban populations listed are those of the selected urban communes; the rural populations are those of the communes falling within the specified 50km radius of that urban area. "With FP" indicates family planning is available through CHWs.

**Definition of Induced Abortion**

For each recalled abortion, the interviewed woman was asked to describe whether the abortion was spontaneous and whether anything had provoked its occurrence. She was then asked to describe what, if anything, had provoked the abortion, and whether any complications had resulted. For the main analysis, all abortions that were described both as "induced" and as "not spontaneous" were considered to be induced abortions. Those that were described as either both or neither "induced" nor "spontaneous" or for which the woman responded that she did not know if it was spontaneous were removed for unclear classification. All women with unclear abortion history (due to at least one unclear abortion, unless another reported abortion was clearly induced) were similarly excluded from all analyses. To assess the robustness of the results to this treatment of abortions with uncertain classification, two sensitivity analyses were run. For the first sensitivity analysis, we considered all abortions described as "induced" as induced, regardless of whether they were also described as "spontaneous", and all other abortions as spontaneous. For the second, we considered all abortions described as both "induced" and "not spontaneous" as induced, and all other abortions as spontaneous.

**Number of subjects**

A target sample size was set to at least 17,100 individuals (19 regions with 30 fokontany each with at least 30 individuals each). Assuming that 17% of these individuals were women of the target age^1^, this would result in 2907 women aged 18-49 being assessed for inclusion. If 20% of these women either were absent, did not provide consent, or were not sexually active, a total of 2,325 women would be included. Assuming an unsafe abortion rate estimates at 0.03/year^2^, and 10 years of follow-up per women, a total of 602 abortions would be expected. With a design effect of 2.0 and a risk of abortion complications of 25%, the precision around this estimate would be approximately 5%.

**Definition of More Effective vs. Less Effective Contraceptive Methods**

We defined "more effective" contraceptive methods to include those listed as "very effective" or "effective" as commonly used by the 2018 WHO Family Planning Global Handbook for Providers^3^ namely implants, male and female sterilization, IUDs, LAM, injectable contraception, and oral contraception. We defined "less effective" contraceptive methods as those listed as "moderately effective" or "less effective" (male and female condoms, the rhythm method, withdrawal, diaphragms, and spermicides) as well as those not listed (abstinence without additional specification, traditional contraceptives, and emergency contraceptives).

**Statistical Analyses**

*Sampling Weights and Design*

Population estimates related to the frequency of abortion, use of specific abortion methods and providers, and occurrence of abortion complications were calculated using the R package "survey" for complex survey analyses. To specify the survey design, we set district and urban/rural setting as strata and fokontany as clusters in the function svydesign. Because the targets for each fokontany were based on the overall number of people censused, not just women in target age range, we similarly treated all individuals as the surveyed population in specifying the survey design and sampling weights. We then subset the survey design object to our population of interest, i.e. sexually active women with identifiable positive or negative history of abortion in the last 10 years. Estimates per abortion were similarly subset from a dataset initially including all surveyed individuals and all abortions.

Inverse probability of selection sampling weights were assigned to each censused individual based on the total stratum population size, the number of fokontany sampled per stratum, and the number of individuals sampled per fokontany per draw. Weights based on the population size of each fokontany were assumed to cancel out based on the probability proportional to size sampling scheme. Values of the weights ranged from as low as 7.6 in rural Mitsinjo (population 8,172) to as high as 1385 in Antananarivo (population 1,247,025).

$$SW = \frac{Pop size of region}{(People sampled per fok draw)*(\# fok draws per region)}$$

Analyses based on univariate and multivariate logistic regression suggested that, among women in the target age range, non-response rates due to absence or lack of consent varied based on district and age but not urban/rural status. Using the predicted values from a multivariate logistic regression without interaction terms, response probabilities for each woman were assigned based on her district and age class (<25, 25 to <35, or ≥35), and response weights defined based on the inverse of the response probabilities. Values of the response weights ranged from 1.1 to 1.9. The sampling weights and response weights were then multiplied to assign a final weight to each woman included in the study.

*Incidence Calculation*

A survey weighted quasi-Poisson regression (function svyglm with family=quasipoisson) was used to estimate the incidence rate of induced abortions. For this calculation, we specified an offset term as the natural log of the minimum of either 10 years or the number of years since initiation of sexual activity. The incidence rate was taken to be the exponent of the intercept term. The dependent variable was the number of induced abortions in the last 10 years per women and there was no independent variable besides the offset.

*Comparisons by Region*

To determine whether regions with family planning (FP) available through CHWs had any differences from those without in terms of self-reported abortion history and contraceptive use, we estimated the proportion of women in rural areas with FP, rural areas without FP, and urban areas reporting a history of abortion in the last 10 years, a history of ever using effective contraceptive methods, and a history of ever using potentially ineffective contraceptive methods.

We then compared separately rural areas without FP and urban areas to rural areas with FP using bivariate design-based Wald tests of association with the function svychisq to see whether the results in rural areas with FP were significantly different from either those in rural areas without FP or urban areas.

*Factors Associated with History of Abortion*

Logistic mixed effects models were used to explore associations between individual-level factors and occurrence of an abortion within the last 10 years. In particular, we specified random effects based on district and fokontany as well as fixed effects for urban/rural status, the number of years at risk, i.e. min(10, years since start of sexual activity). Our “unadjusted” models contained also only the variable of interest. Our “adjusted” model contained all of the variables described in the table below. The dependent variable was a binary variable for having had an induced abortion in the last 10 years (1=yes, 0=no). In a sensitivity analysis, adding a random effect for household had negligible impact on the results. Calculations were performed using the R function glmer from package lme4.

Using this framework, we tested sequentially the following individual-level variables for any possible association with history of abortion (Table S2): age, highest level of education attained, religion, civil status, socioeconomic status (based on quintiles of all interviewed women), history of transactional sex (whether the woman reported ever receiving money/gifts/favors in exchange for sex), history of live births, desired number of children (below the median of 4 or not), and ever having used contraception (ever used potentially ineffective methods, ever used effective methods only, or never used).

Table S2: Categorization of independent variables included in analysis of risk factors for history of abortion

| Variable | Values |
| --- | --- |
| Age | ≥35 years |
|  | 25 to <35 years |
|  | 20 to <25 years |
|  | <20 years |
| Maximum Education | Primary school or less |
|  | Middle school |
|  | High school or more |
| Religion | Catholic |
|  | Church of Jesus Christ in Madagascar (FJKM) |
|  | Other Christian |
|  | Other (including Muslim, traditional religions) |
| Civil Status | Single (never married) |
|  | Married or living with a partner |
|  | Other (widowed, divorced) |
| Socioeconomic status, according to socioeconomic index developed based on assets and amenities | Quintile 1 |
|  | Quintile 2 |
|  | Quintile 3 |
|  | Quintile 4 |
|  | Quintile 5 |
| Transactional Sex | No (never received money/gifts/favors in exchange for sex) |
|  | Yes (ever received money/gifts/favors in exchange for sex) |
| Number of Previous Live Births | 0 previous live births |
|  | ≥1 previous live births |
| Ideal Number of Children | <4 |
|  | ≥4 |
| Contraceptive Use | No history of contraceptive use |
|  | History of using less effective methods only |
|  | History of using more effective methods only |
|  | History of using both more and less effective methods |

*Factors Associated with Abortion Complications and Care Seeking Behavior*

We also assessed the relationship between abortion methods and providers (independent variables) and the occurrence of complications following the abortion (dependent variables). Abortion methods were dichotomized to "misoprostol alone" (oral and/or vaginal misoprostol, but not any other methods) and "not misoprostol alone" (any other or unknown methods, regardless of whether misoprostol was used). Abortion providers were dichotomized to "qualified medical personnel only" (nurse, doctor, and/or midwife only) and "not only qualified medical personnel (may also include herself, traditional birth attendants or healers, or unspecified other personnel).

The outcomes considered here were binary variables for potential infection following the abortion (based on self-reported fever, chills, or foul-smelling vaginal discharge) and seeking care for complications following the abortion. These analyses were also performed using logistic mixed effects models, with random effects based on district, fokontany, and individual, and fixed effects for urban/rural status. These calculations were also performed using the R function glmer. All calculations were performed conditional on the month of pregnancy at which the abortion was performed. The variables for abortion provider and method were considered in both separate and combined models; the results did not differ in terms of significance, and thus only the full multivariate model results are presented.

**Results: Month of Pregnancy**

**Fig S1: Month of pregnancy that the abortion occurred**

**Results: Care seeking for complications of abortion**

| **Care-Seeking Behavior** | **Percentage of All Abortions** |
| --- | --- |
| Sought treatment | 27.7% (21.8, 34.6) |
| Hospitalized | 2.4% (1.1, 5.4) |
| **Individual or Location Consulted** | **Percentage of Abortions for Which Treatment Is Sought** |
| Self-treatment | 31.9% (17.9, 50.3) |
| Family or Friend | 22.9% (13.4, 34.5) |
| Public Hospital or Health Center | 28.0% (18.5, 40.1) |
| Private Hospital or Clinic | 56.6% (42.5, 69.6) |

**Table S3: Care seeking after abortion.** Women could list up to three places that care was sought per abortion. Zero women reported seeking care from a pharmacy, traditional healer or birth attendant (matrone), or CHW.

**Results: District-level accessibility of contraception**

Women in rural areas where family planning is provided by community health workers appeared less likely to have experienced induced abortion than women in either other rural areas (weighted chi-squared p=0.0005) or women in urban areas (p=0.0001) (Table S4). These women were also more likely to have ever used an effective contraceptive method^18^ than women in other rural areas (p<0.0001) or women in urban areas (p=0.0002). They tended to be less likely to have used a potentially ineffective contraceptive method, though this association was only significant compared to urban women (p=0.007) and not rural women from other areas (p=0.11).

|  | Induced Abortion | More Effective Contraceptive Method: Ever Used | Less Effective Contraceptive Method: Ever Used |
| --- | --- | --- | --- |
| Rural with FP | 3.9% (2.5– 6.0) | 73.9% (69.2– 78.1) | 11.2% (8.8–14.2) |
| Rural without FP | 9.9% (7.4–13.1) | 56.0% (50.2–61.6) | 15.4% (11.4–20.4) |
| Urban | 12.7% (9.1–17.4) | 58.2% (51.6–64.4) | 19.4% (14.8–25.1) |

**Table S4: Induced abortions and use of contraception in areas with and without family planning.** FP: family planning provided by community health workers. See supplemental methods for detailed definitions of effective and ineffective contraceptive methods.

**Sensitivity analysis**

For the first sensitivity analysis scenario, we treated all abortions that were reported as "induced" to be induced abortions, regardless of whether they were also described as spontaneous, and all others to be not induced. For the second, we treated all abortions that were reported as "induced" and "not spontaneous" to be induced, and all others to be not induced. Results were largely consistent with those reported for the main analysis, including an incidence rate of induced abortion of 20.2 per 1000 person-years at risk in the first scenario and 17.7 per 1000 person-years in the second. Though the magnitudes and directions of the regression results remained largely similar, the first scenario reduced the strength of the (individual) associations with contraceptive use to an odds ratio of 1.31 (p=0.25) for less effective methods, 1.58 (p=0.007) for more effective methods, and 2.98 (p<0.0001) for both (results of multivariate model).

**References**

1. Institut National de la Statistique. Enquête Démographique et de Santé de Madagascar 2008-2009. Antananarivo, Madagascar, 2010.

2. Sedgh G, Bearak J, Singh S, et al. Abortion incidence between 1990 and 2014: global, regional, and subregional levels and trends. *Lancet* 2016; **388**(10041): 258-67.

3. World Health Organization Department of Reproductive Health and Research (WHO/RHR) and Johns Hopkins Bloomberg School of Public Health/Center for Communication Programs (CCP) Knowledge for Health Project. Family planning: a global handbook for providers (2018 update). Baltimore and Geneva, 2018.
